# Nestimate: An R Package for dynamic, probabilistic, and high order network analysis

**DOI:** 10.1177/01466216261472019

**Published:** 2026-07-26

**Authors:** Mohammed Saqr, Sonsoles López-Pernas, Kamila Misiejuk

**Affiliations:** 1 163043 School of Computing, University of Eastern Finland, Joensuu, Finland; 2 26553 CATALPA, FernUniversitat in Hagen, Hagen, Germany

**Keywords:** Nestimate, network estimation, psychological networks, factor analysis, higher-order networks, R package

## Abstract

Networks are often estimated from data such as psychometric scales, event data, and ecological momentary assessment data. We introduce the Nestimate R package, which offers a unified interface for estimating networks through a standardized workflow. First, Nestimate estimates psychological network models using correlation, partial-correlation, regularized (graphical lasso), Ising, and mixed graphical networks from both frequency and event data. Second, Nestimate introduces multi-cluster networks, which aggregate psychometric networks using, for example, factor loadings, principal components, or network connectivity. Third, higher-order network models represent dynamics whose probability depends on more than the immediately preceding state and offer a rich interface for measuring and evaluating higher-order behaviors such as critical thinking. Fourth, Nestimate provides functions for estimating dynamic networks, including transition, co-occurrence, and mixed networks, for capturing behavioral dynamics from event data. Every estimated network can be subjected to a unified validation pipeline, including non-parametric bootstrapping, permutation testing, split-half reliability, and case-dropping centrality stability analyses.

## Description of the Package

Networks are increasingly used to study relationships, dependencies, and dynamics in psychological, behavioral, and longitudinal data. We introduce the Nestimate R package, a unified framework for estimating, validating, and comparing network models (Saqr et al., 2025a). Nestimate supports psychological networks, including correlation, partial-correlation, graphical lasso, Ising, and mixed graphical models; dynamic networks derived from event and sequence data, such as transition, frequency, attention-weighted, co-occurrence, and mixed networks; cluster- and group-based network approaches that identify heterogeneous patterns through sequence clustering, mixed Markov models, and multi-cluster representations; and higher-order network models that capture dependencies extending beyond immediate transitions through higher-order networks, embeddings, anomaly detection, and multi-order generative models (Epskamp & Fried, 2018; Xu et al., 2016). In addition, Nestimate provides tools for multilevel vector autoregressive models, idiographic network estimation, topological network analysis, link prediction, and longitudinal network modeling. Beyond network estimation, the package includes a broad suite of sequence, pattern, and descriptive analytics for exploring behavioral processes. These include sequence description and visualization, clustering, and temporal characterization of learning and behavioral pathways (Saqr et al., 2025b). The Nestimate package (Saqr et al., 2025a) estimates networks from event data, psychometric scales, and frequency data through a unified interface in R (R Core Team, 2024).

A central feature of Nestimate is a unified validation framework that can be applied across network models following the Dynalytics framework (Saqr et al., 2026). This framework includes suitability analyses; split-half reliability analyses for assessing the consistency and replicability of network structures across random data partitions; case-dropping procedures that evaluate the robustness of network properties as progressively larger proportions of observations are removed; non-parametric bootstrapping techniques for determining whether individual edges and transitions reflect systematic patterns rather than chance; and representational validity analyses that quantify the structural distortions introduced by row-normalization and related transformations at the edge level.

## Availability, Documentation, and Distribution

The Nestimate package, documentation, and the supplementary materials are available from the Comprehensive R Archive Network (https://CRAN.R-project.org/package=Nestimate) and GitHub (https://github.com/mohsaqr/Nestimate/). The supplementary materials include the complete documentation of the package features and provide illustrative examples and tutorials on the practical use of the package, covering transition networks, psychological networks, multi-cluster aggregation, and higher-order networks. The documentation and tutorials can also be viewed online on the package website (https://pak.dynasite.org/Nestimate/).
